# A staff support programme for rural hospitals in Nepal

**DOI:** 10.2471/BLT.15.153619

**Published:** 2015-11-02

**Authors:** Mark Zimmerman, Sharada Shah, Rabina Shakya, Bal Sundar Chansi, Kashim Shah, Daniel Munday, Nir Eyal, Bruce Hayes

**Affiliations:** a Nick Simons Institute, Box 8975, EPS 1813, Kathmandu, Nepal.; b Division of Health Sciences, Warwick University, Warwick, England.; c Harvard TH Chan School of Public Health, Harvard University, Boston, United States of America.

## Abstract

**Problem:**

District hospitals in Nepal struggle to provide essential services such as caesarean sections.

**Approach:**

Retention of health workers is critical to the delivery of long-term, quality health-care services. To promote retention and enhance performance in rural public hospitals, the Government of Nepal and the Nick Simons Institute progressively implemented a rural staff support programme in remote hospitals. After competitive selection for a compulsory-service scholarship and training, family practice doctors who could do basic surgery, orthopaedics and obstetrics were hired under a binding three-year contract in each participating hospital. Comfortable living quarters and an Internet connection were provided for the resident doctors; in-service training for all staff and capacity development for each hospital’s management committee were provided.

**Local setting:**

Nepal’s mountainous landscape, poverty and inequitable rural/urban distribution of health workers pose barriers to adequate health care.

**Relevant changes:**

Between 2011 and 2015 family practice doctors were maintained in all seven programme hospitals. All hospitals became providers of comprehensive emergency obstetric care and served more patients. Compared with hospitals not within the programme, deliveries increased significantly (203% versus 71% increase, respectively; *P* = 0.002). The programme recently expanded to 14 hospitals.

**Lessons learnt:**

A package of human resource supports can improve the retention of doctors and the use of remote hospitals. Factors contributing to the success of this programme were compulsory-service scholarship, central personnel management, performance-based incentives and the provision of comfortable living quarters.

## Résumé

**Problème:**

Les hôpitaux de district du Népal ont du mal à fournir des services essentiels tels que des césariennes.

**Approche:**

Le maintien des professionnels de santé s'avère crucial pour pouvoir fournir des services médicaux de qualité sur le long terme. Afin d'améliorer ce maintien ainsi que les performances des hôpitaux publics situés en zone rurale, le gouvernement népalais et le *Nick Simons Institute* ont progressivement mis en place un programme de soutien du personnel dans les hôpitaux reculés. Après une sélection par concours pour bénéficier d'une formation et d'une bourse conditionnée à un service obligatoire, des médecins généralistes pouvant réaliser des actes basiques dans le domaine chirurgical, orthopédique et obstétrique ont été recrutés dans chaque hôpital participant au titre d'un contrat de trois ans. Un logement confortable et une connexion Internet ont été fournis aux médecins résidents; dans chaque hôpital, l'ensemble du personnel a bénéficié d'une formation continue et le comité de gestion d'un renforcement des compétences.

**Environnement local:**

Le relief montagneux du Népal, la pauvreté et la répartition inéquitable des professionnels de santé entre milieu rural / urbain sont autant d'obstacles à l'offre de soins adéquats.

**Changements significatifs:**

Entre 2011 et 2015, les médecins généralistes sont restés dans l'ensemble des sept hôpitaux du programme. Tous ces hôpitaux ont pu délivrer des soins obstétricaux d'urgence et prendre en charge davantage de patients. Comparés à des hôpitaux ne faisant pas partie du programme, les soins dispensés ont considérablement augmenté (augmentation de 203% contre 71%, respectivement; *P* = 0,002). Le programme a récemment été étendu à 14 hôpitaux.

**Leçons tirées:**

Des dispositifs de soutien des ressources humaines peuvent améliorer le maintien des médecins et le recours aux hôpitaux situés dans des zones reculées. Les facteurs qui ont contribué à la réussite de ce programme sont la bourse conditionnée à un service obligatoire, la gestion centralisée du personnel, les primes au rendement et la mise à disposition de logements confortables.

## Resumen

**Situación:**

Los hospitales de distrito en Nepal tienen dificultades para proporcionar servicios básicos como cesáreas.

**Enfoque:**

La retención del personal sanitario es indispensable para suministrar servicios médicos de calidad a largo plazo. Para promover la retención y mejorar el rendimiento en los hospitales públicos rurales, el Gobierno de Nepal y el Instituto Nick Simons aplicaron de forma progresiva un programa de apoyo al personal rural en hospitales remotos. Tras una selección competitiva para una beca de servicio obligatorio y formación, se contrató en cada hospital participante a médicos de familia que pudieran llevar a cabo una cirugía básica, ortopedias y obstetricia con un contrato vinculante de tres años. Se proporcionó una cómoda residencia y conexión a internet a los médicos residentes y también formación en el servicio para todo el personal y desarrollo de capacidades para el comité de gestión de cada hospital.

**Marco regional:**

En Nepal, el paisaje montañoso, la pobreza y la injusta distribución del personal sanitario entre las zonas rurales y urbanas obstaculizan una adecuada atención sanitaria.

**Cambios importantes:**

Entre 2011 y 2015 se mantuvieron médicos de familia en los siete hospitales del programa. Todos los hospitales se volvieron proveedores de atención obstétrica de emergencia integral y atendieron a más pacientes. En comparación con otros hospitales que no se encontraban en el programa, el número de partos aumentó significativamente (aumentó del 203% frente al 71%, respectivamente; *P* = 0,002). Recientemente, el programa se extendió hasta los 14 hospitales.

**Lecciones aprendidas:**

Un paquete de apoyo a los recursos humanos puede mejorar la retención de médicos y el uso de hospitales remotos. Los factores que contribuyeron al éxito de este programa fueron la beca de servicio obligatorio, la gerencia central de recursos, los incentivos basados en el rendimiento y la provisión de una residencia cómoda.

## ملخص

**المشكلة:**

تبذل المستشفيات بالأقاليم في نيبال جهودًا مضنية لتوفير الخدمات الضرورية مثل خدمات أقسام الجراحات القيصرية.

**الأسلوب:**

إن الاحتفاظ بالعاملين في مجال الخدمات الصحية يمثل أمرًا شديد الأهمية لتقديم خدمات الرعاية الصحية بمستوى جيد على مدى طويل. وتولت حكومة نيبال ومعهد نيك سايمونز تنفيذ برنامج لتقديم الدعم تدريجيًا للعاملين بالمناطق الريفية في المستشفيات بالمناطق النائية، وذلك لتحسين مستوى الاحتفاظ بالعاملين ورفع مستوى الأداء بالمستشفيات العامة بالمناطق الريفية. وبعد إجراء انتقاء يعتمد على التنافسية للحصول على منحة دراسية وتدريب يختص بالخدمات الإلزامية، تم تعيين أطباء ممارسين لطب الأسرة العام ممن يمكنهم إجراء الجراحات الأساسية، وتقديم العون فيما يتعلق بطب العظام وأمراض النساء والتوليد بموجب عقد ملزم لمدة ثلاث سنوات في كل مستشفى مشاركة في هذه التجربة. وتم توفير أماكن مريحة للإقامة واتصال بالإنترنت للأطباء المقيمين، وتدريب بالممارسة لجميع العاملين، كما تم تقديم سبل تنمية القدرات لكل لجنة من لجان التنظيم الإداري بالمستشفيات.

**المواقع المحلية:**

إن الطبيعة الجبلية في نيبال والفقر والتوزيع غير العادل للعاملين بمجال الخدمات الصحية في المناطق الريفية/الحضرية تضع بعض العقبات في سبيل تقديم خدمات الرعاية الصحية.

**التغيرات ذات الصلة:**

تم الاحتفاظ بالأطباء الممارسين لطب الأسرة العام في الفترة بين عامي 2011 و2015 في جميع المستشفيات التي شملها برنامج اختص بسبع مستشفيات. وأصبح الكل في تلك المجموعة من المستشفيات من مقدمي الرعاية الصحية الشاملة لحالات التوليد الطارئة، كما قدموا خدماتهم للمزيد من المرضى. ومقارنةً بالمستشفيات التي لم يشملها البرنامج، فقد زادت نسبة الولادة بها بدرجة ملحوظة (حيث بلغت الزيادة 203% مقابل 71%، على التوالي؛ (الاحتمال = 0.002). واتسع نطاق البرنامج ليشمل 14 مستشفى مؤخرًا.

**الدروس المستفادة:**

يمكن لمجموعة من حالات دعم الموارد البشرية أن ترفع مستوى الاحتفاظ بالأطباء والاستفادة من المستشفيات بالمناطق النائية. وتمثلت العوامل المساهمة في نجاح هذا البرنامج في المنح الدراسية المختصة بالخدمة الإلزامية، والإدارة المركزية لشؤون العاملين، والحوافز المعتمدة على الأداء، وتوفير أماكن مريحة للإقامة.

## 摘要

**问题:**

尼泊尔地方医院难以提供基本医疗服务（如剖腹产）。

**方法:**

医疗工作者的留任对长期提供高质量医疗保健服务至关重要。为了促进乡村公立医院的人才留任，并提高其绩效表现，尼泊尔政府和尼克西蒙研究所 (Nick Simon Institute) 逐步落实偏远乡村医院人力支持计划。每所参与该计划的医院都可与通过奖学金（附带强制服务要求）和培训机会竞选，且有能力进行基本外科手术、整形手术和助产手术的家庭执业医师签订为期三年的合约。该计划还将为住院医师提供舒适住所和网络连接、为所有人员提供在职培训，并且为每所医院的管理委员会提供能力拓展服务。

**当地状况:**

尼泊尔地区地处山区、物资匮乏且城乡医疗工作者分配不均，阻碍了医疗护理资源的合理配置。

**相关变化:**

2011 年至 2015 年间，所有参与计划的医院（共七所）内的家庭执业医师全部继续留任。所有医院都能提供综合性产科紧急护理，并且有能力服务更多患者。参与该计划的医院所进行的分娩数量与未参与该计划的医院相比，有了显著增长（增长率分别为 203% 和 71%；P=0.002）。该计划近期已扩展至 14 所医院。

**经验教训:**

一整套的人力资源支持可增加医生的留任率和偏远医院的利用率。促成该计划的因素包括：附带强制服务要求的奖学金、集中化人事管理、根据绩效展开的激励制度和提供舒适住所。

## Резюме

**Проблема:**

Районные больницы Непала испытывают трудности при оказании важнейших услуг населению, таких как выполнение кесарева сечения.

**Подход:**

Удержание работников на их рабочих местах крайне важно для оказания продолжительных услуг здравоохранения высокого качества. Чтобы поощрять медработников оставаться на рабочих местах и улучшить показатели качества работы сельских больниц, правительство Непала и Институт Ника Саймонса последовательно осуществили программу поддержки персонала больниц, расположенных в отдаленной местности. Среди врачей общей практики, умеющих выполнять хирургические операции общего характера, ортопедические операции и осуществлять акушерскую помощь, был проведен конкурсный отбор с условием получения дотации и обучения в обмен на обязательство отработать определенный срок; прошедший такой отбор специалист подписывал трехлетний обязательный контракт на работу в одной из больниц, участвовавших в конкурсе. Врачам, проживающим в данной местности, предоставлялось комфортабельное жилье и канал доступа в сеть Интернет; весь персонал больницы проходил обучение без отрыва от производства, а для руководящего комитета каждой больницы проводились курсы повышения квалификации.

**Местные условия:**

Непал — гористая страна, в которой нищета и неравномерное распределение медицинских сотрудников в городской и сельской местности создают препятствия для нормального оказания медицинских услуг.

**Осуществленные перемены:**

В период с 2011 по 2015 год врачи общей практики присутствовали во всех семи больницах, охваченных программой. Все больницы стали предоставлять полный спектр срочных акушерских услуг и смогли обслужить большее количество пациентов. По сравнению с больницами, не вошедшими в программу, количество родов значительно возросло (на 203% в сравнении с 71% соответственно; *P* = 0,002). Недавно программу расширили, и теперь в ней участвуют 14 больниц.

**Выводы:**

Комплекс мер по поддержке кадров способствует удержанию врачей на их рабочем месте, а также улучшает показатели обращений в больницы, расположенные в отдаленной местности. Факторами успеха программы были признаны: денежное вознаграждение за обязательство отработать определенный срок в сельской местности, централизованное управление персоналом, поощрения, зависящие от показателей качества работы, и предоставление комфортабельного жилья.

## Introduction

In remote areas, an absence of doctors and nurses leads to poor health outcomes for local populations.^1^ To increase access to health-care workers, the World Health Organization recommends interventions in four areas – education, regulatory, financial and professional/personal.^1^ To support retention, WHO and other organizations have called for bundled programmes that take into account health workers’ expectations.^2^^–^^4^ Retention programmes that enhance workers’ competence, responsiveness and productivity have also been recommended.^5^

Studies on retention of health-care workers in low- or middle-income countries tend to focus on compulsory government service^6^ or on salary incentives.^7^^,^^8^ Few studies have reported on bundled programmes or used patient volumes as outcome variables for such programmes.^3^^,^^6^^,^^8^

Here we describe a bundled programme for human resource support in Nepal.

## Local setting

In Nepal, the mountainous landscape, poverty (the annual gross domestic product per capita is 300 United States dollars, US$) and an inequitable rural/urban distribution of health workers pose barriers to adequate health care.^9^^,^^10^

Eighty-three percent of 28 million Nepalese live in rural areas. These areas are served by 15-bed public district hospitals,^11^ which are expected by the Nepalese Government to provide emergency operations. However, in 2006, only 10 of 64 (16%) district hospitals were able to perform caesarean sections, due to absent, low-performing or mismatched health-care workers.^12^

## The support programme

To address low retention of health-care workers and poor performance in district hospitals, the Nepalese Government partnered in 2006 with the Nick Simons Institute – a nongovernmental organization working to improve health care in rural areas by supporting Nepalese health-care workers. The partnership developed a rural staff support programme, based on international consensus about retention factors,^1^^,^^3^ experience in the Nepalese health-care system^13^ and stakeholder consultations.

The central component of the rural staff support programme was recruiting one or two family practice doctors per programme hospital. These physicians are post-graduate doctors trained in medical universities in Nepal – to provide primary care as well as basic surgery, orthopaedics and obstetrics. To recruit these doctors, we first negotiated with the medical university for three to six seats per year in the post-graduate family practice programme. Then we advertised in newspapers for junior doctors with at least two years of working experience and who had either been raised in or who had previously worked in rural areas. We chose 15 to 20 applicants to take an entry exam. Three to six applicants with the best results were offered a scholarship for the three-year post-graduate programme and binding contracts for a subsequent three years of service in a programme hospital. Doctors who chose to leave the programme early incurred a financial penalty twice the scholarship, which varied from US$ 20 000 – 30 000. Once posted in the programme hospital, the doctors received salaries three times higher than the usual basal government rate, not including other government benefits. To facilitate an effective hospital team, the programme also provided personal, professional and management support for all staff working in the hospital (Table 1).

**Table 1:** Overview of the rural staff support programme, Nepal, 2007–2015

Support	Description	First phase assessment (2010)	WHO policy recommendation category^a^
**Original**			
Clinical coordination by family practice doctor	Employ two family practice doctors past their scholarship commitment	Most critical component to increasing hospital use	A1, B4, C1, D2, D6
Comfortable quarters	Build new and renovate existing doctors’ quarters	Appreciated, but staff also requested the same improvements for all staff quarters	D1
Communication	Provide reliable Internet access in quarters and hospital office	Important component for reducing sense of isolation	D1, D5
Continuing medical education	Train multiple levels of staff – via in-service courses and on-the-job trainings	Encouraging to all staff; special value for quality of delivery service	A5, D3
Community governance	Participate in and build capacity of local hospital management committee	Variable ownership by different local committees	D2
Capital items	Procure equipment or do small building projects to improve clinical services	Important for starting medical procedures, such as operations	D2
**Discontinued (2007–2011)**			
Children’s education support	Assist two primary schools located near to the hospital	Discontinued: no trickle down to hospital performance	D1
Connection with larger hospital	Partner smaller programme hospital with a mentor hospital in the region	Discontinued: larger hospitals too busy to assist district hospitals	D6
**Added (2011–2015)**			
Connection with district	Develop training and referral linkage with smaller district health posts	The programme should evolve towards district-wide support	–
Continuous quality improvement	Initiate and monitor an ongoing cycle of self-assessment and interventions	Performance improvement should affect service quality and not just utilization	D2

WHO: World Health Organization.

^a^ The categories of WHO improved retention policy recommendation are as follow: A: education; B: regulatory; C: financial; D: professional/personal.^1^

The programme was implemented stepwise: In 2007, three hospitals started the programme and in 2009, they were fully operational when scholarship doctors began to graduate from their training programmes. In 2011, four more hospitals joined the programme. All seven hospitals (Bajhang, Kalikot, Doti, Salyan, Kapilvastu, Gulmi and Dolakha) were rural, some were in extremely mountainous regions, and all their districts were below the national Human Development Index mean of 0.471.^14^

In 2008, we hired one centrally-located nurse coordinator for the programme team. This nurse administered the programme and provided counselling to doctors and nurses posted in the remote hospitals. She shared success stories with other hospitals, the government, the local newspaper and published these in the institute’s annual reports. When the programme grew to seven hospitals, we hired a second nurse coordinator.

In 2010, we did a detailed evaluation. Hospital staff mostly appreciated the Internet access, hospital equipment stipends and renovations of staff quarters. However, two components did not produce the expected outcomes. The children’s education component assisted certain schools, yet many staff chose to send their children to other schools. Efforts to connect these small rural hospitals with a larger hospital failed because staff in the larger hospitals were too busy with their own work to develop useful support relationships with staff working in the programme hospitals. These two components were therefore replaced with two new components in 2011 – connection with district health posts and continuous quality improvement (Table 1). We also interviewed a small number of relatives of outpatients and inpatients to assess community satisfaction.

In 2011 we added a job description for the family practice doctors and a performance-based incentive, which was a lump sum of maximum 20% of the total salary. The lump sum depended on a doctor evaluation score. For example, if the doctor scored 80%, he or she got 80% of the maximum lump sum. In each hospital, we also introduced and monitored a self-administered quality improvement tool designed to address equipment and management gaps that affected patient care.

The institute bore all programme costs. The programme cost was US$ 66 387 per hospital per year – 49.2% (US$ 32 667) for doctors’ scholarships and salaries, 45.3% (US$ 30 073) for other programme activities and 5.5% (US$ 3651) for central management. This cost was approximately 50% more than a parallel, government scheme that provided emergency obstetric services through one-year doctor contracts in 28 district hospitals.^15^

## Programme outcomes

All doctors reported that they settled uneasily into their new workplaces: they felt isolated, both geographically from their homes and professionally from the medical hierarchy of their training hospitals. Nevertheless, the programme was able to continuously post at least one family practice doctor in each of the seven hospitals.

Five out of the programme’s first 20 doctors chose to pay off their bond before fully completing their service period. As of 2013, three of these five doctors continued to work in rural hospitals for other organizations.

All seven programme hospitals became providers of emergency obstetric care and doctors did between 10 and 50 caesarean sections per year in each hospital.

We used changes in hospital use as a proxy indicator for community satisfaction and quality of care. We collected data on numbers of outpatient visits, admissions, deliveries and caesarean sections. Comparing patient use before (2006–2007) and after (2012–2013) implementing the programme, all programme hospitals showed increases in all four indicators. Mean annual admissions and outpatient visits per hospital almost doubled, from 832 to 1592, and from 10 585 to 21 341, respectively. Mean deliveries per hospital per year tripled, from 152 to 462. The mean annual increase in caesarean sections was 23.4 per year; from 1.4 to 24.8.

**Fig. 1:**
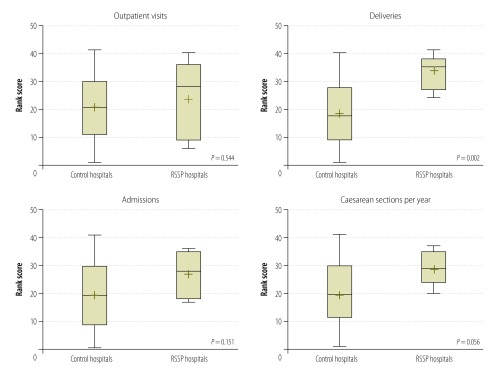
Changes in hospital use ranks in control and rural staff support programme hospitals, Nepal, 2007–2013

We compared use data between programme hospitals and district hospitals not within the programme. The 34 control hospitals with complete data also showed increased use between 2006 and 2013. Mean annual admissions, outpatient visits and deliveries per hospital increased approximately 1.5 times, from 1231 to 1770, from 13 065 to 19 299, and from 298 to 511, respectively. The mean annual increase in caesarean sections increased from 2.1 to 24.5. When compared to the control hospitals – using a non-parametric rank test – programme hospitals had greater improvements in the number of deliveries (*P* = 0.002) and caesarean sections (*P* = 0.056). Admissions (*P* = 0.151), and outpatient visits were not significantly increased (*P* = 0.544; Fig. 1).

RSSP: rural staff support programme.

Notes: Control and programme hospitals’ ranks were compared using a Wilcoxon Rank Sum test. The mean rank is indicated with a plus sign (+) and the horizontal bar indicates the median rank. The box represents the 25th and 75th percentiles of the indicator and the whiskers extend to largest or smallest values, excluding outliers. Seven rural staff support programme hospitals and 34 control hospitals were included in the analysis.

In the evaluation and during regular hospital visits, staff strongly requested that the programme be continued in their hospital. Though staff other than doctors appreciated the programme, they complained that they were not being compensated for having to treat more patients.

## Lessons learnt

To improve performance in poorly functioning rural public hospitals, we created a staff placement and support programme based on three principles – personalized management, bundled support and programme evaluation and revision.

We recruited and deployed family practice specialists who were capable of providing a range of services. We added several comfort and professional supports to a bundle that we revised after evaluation to improve the programme. The use of and types of surgical services available in the programme hospitals increased (Box 1). Compared with control hospitals, all programme hospitals showed a higher increase in deliveries; all hospitals were converted into continuous providers of emergency obstetrics services; and the changes in hospital services all met with community satisfaction. While a parallel government-contracted doctor programme provided discontinuous service,^15^ the rural staff support programme maintained a continuous supply of family practice doctors to each hospital.

Box 1 **Summary of main lessons learnt**

- Continuous doctor retention in remote, previously understaffed hospitals can be attained through a combination of compulsory service scholarship, improved living quarters, personal counselling and moderately higher salaries, including performance-based incentives.
- Programme success depends on the performance of the individual doctor – which in turn depends on both the doctor’s personal qualities – such as courage and motivation – and a range of hospital factors.
- Hospital staff found Internet access, new equipment and comfortable quarters especially helpful.

The main challenge of the programme was to motivate – and ensure effective collaboration between – family practice doctors and their government medical superintendents. We had to overcome the preconceptions that rural hospitals had always been, and would likely remain, dysfunctional. This challenge was addressed by the programme’s long-term commitment and its responsiveness to each hospital’s unique situation. Another challenge was the medical superintendents themselves, who varied widely in both their desire to upgrade their own hospitals and their management capacity. We addressed this challenge by giving them control over the equipment and training budgets and giving them credit for the programme gains.

In 2013, during the sixth year of the programme, the Nepalese government asked that the programme be expanded to 25 more district hospitals. However, limited numbers of scholarship doctors meant that only four hospitals in 2013 and three in 2015 could join the programme, resulting in a total of 14 functioning programme hospitals. The programme plans to expand to 18 hospitals. In 2015, the Nepalese health ministry, using its own budget, instituted a similar programme to recruit, bond and deploy 40 family practice doctors per year in public hospitals.

A compulsory-service scholarship programme for doctors that includes staff and living supports could be a viable model in other countries that face problems in delivering health-care services in remote areas.
